# Use of Tofacitinib for infant-onset STING-associated vasculopathy: A case report from China

**DOI:** 10.1097/MD.0000000000031832

**Published:** 2022-12-02

**Authors:** Danping Shen, Xiaorui Fan, Qing Zhou, Xuefeng Xu, Meiping Lu

**Affiliations:** a Department of Rheumatology Immunology and Allergy, Children’s Hospital, Zhejiang University School of Medicine, National Clinical Research Center for Child Health, Hangzhou, China; b Life Sciences Institute, Zhejiang University, Hangzhou, China.

**Keywords:** cutaneous vasculopathy, interferonopathies, interstitial lung disease, JAK inhibitor, SAVI

## Abstract

Stimulator of interferon gene (STING)-associated vasculopathy with onset in infancy (SAVI), caused by gain-of-function mutations in human transmembrane protein 173 (*TMEM173*), is characterized by widespread chronic inflammation primarily affecting the skin and lungs. Although SAVI is an inflammatory disease, typical anti-inflammatory agents have limited or no effect.

**Methods and Results:**

A 1-year-old boy presented with recurrent facial rashes since he was 8 months. Moreover, he suffered from recurrent oral ulcers, chronic cough, and failure to thrive. Laboratory parameters showed elevated erythrocyte sedimentation rate (ESR) and immunoglobulin levels. Chest high-resolution computed tomography (HRCT) showed interstitial lung disease (ILD). Whole-exome sequencing revealed a heterozygous mutation in the *TMEM173* gene (c.463G > A, p.V155M). Ultimately, the patient was diagnosed with SAVI. Tofacitinib was initiated at the age of 19 months, resulting in the alleviation of facial rashes and improvement of ILD within 3 months.

**Conclusion:**

SAVI is a difficult-to-treat type I interferonopathy. We hope that JAKi treatment will prove valuable for SAVI patients.

## 1. Introduction

Type I interferonopathies are a newly identified class of disorders linked to the upregulation of type I interferon (IFN).^[1,2]^ Liu et al first described SAVI in 2014. SAVI is caused by heterozygote gain-of-function mutations in *TMEM173*, resulting in constitutive activation of STING and overproduction of type I IFN.^[3]^ Clinically, SAVI is characterized by neonatal-onset systemic inflammation, including severe cutaneous vasculopathy, and ILD.^[4,5]^ Although SAVI is an inflammatory disease, typical anti-inflammatory agents such as corticosteroids, intravenous immunoglobulins, anti-TNF, and anti-CD20 have limited or no effect.^[6]^ The prognosis is poor and death occurs with disease progression in severe cases, especially those with recurrent skin and lung infections and ILD.^[4,7]^

There have been few reports of Janus kinase inhibitor (JAKi) treatment for SAVI. However, results of JAKi treatment in other IFN diseases have been encouraging,^[8]^ and due to its ability to block type I IFN pathway activation in peripheral blood mononuclear cells (PBMCs) in SAVI patients in vitro, good responses to JAKi treatment have been observed.^[9,10]^

We describe a SAVI patient from China who presented with a dark red frostbite-like rash on the face, chronic cough, recurrent oral ulcers, and failure to thrive. Treatment with the JAKi tofacitinib improved the cutaneous manifestations and ILD, but no significant improvement was seen in the oral ulcers or growth rate.

## 2. Case presentation

**Figure 1.**
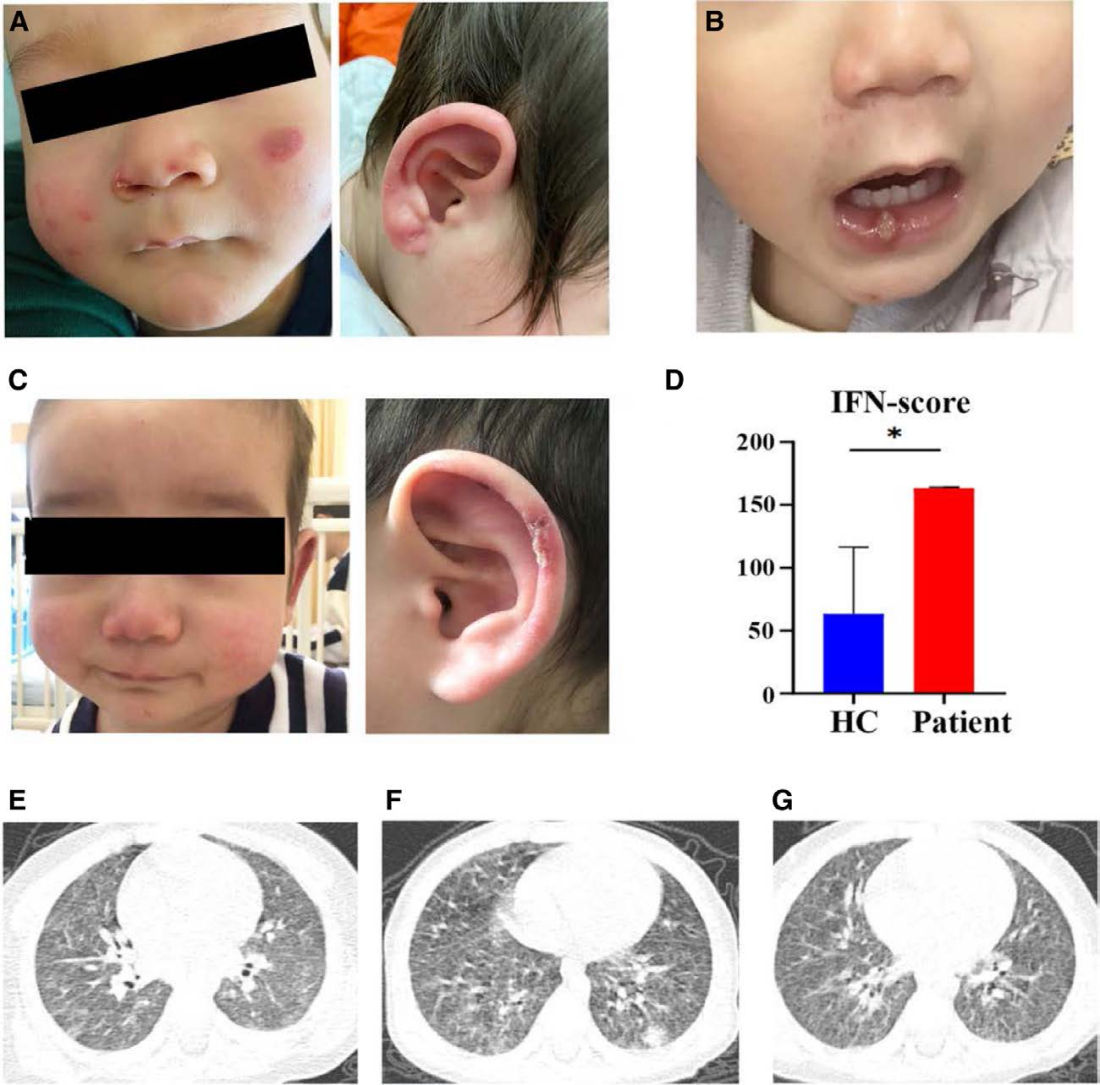
Clinical features of the patient. (A) Photograph of the facial rashes were present on the cheeks, nose tip, and auricles on both sides. (B) Photograph of patient with oral ulcer. (C) Photograph of the facial rashes after treatment of tofacitinib. (D) The expression of IFN regulated genes in health control (HC) and patient. (E–G) Chest CT of patient. CT = computed tomography, IFN = interferon.

A 1-year-old boy, born at full-term with normal weight, height, and head circumference, developed recurrent rashes at 8 months of age. He presented with a frostbite-like rash on the cheeks, nose tip, and auricles, which worsened in the cold and winter. Moreover, he suffered from recurrent oral ulcers (Fig. 1B) and cough in the first year of his life. He also had failure to thrive with a height of 68.5 cm (below the third standard deviation) and weight of 8 kg (between the second and third standard deviations). The family history indicated that his grandfather and father had ankylosing spondylitis.

**Figure 2.**
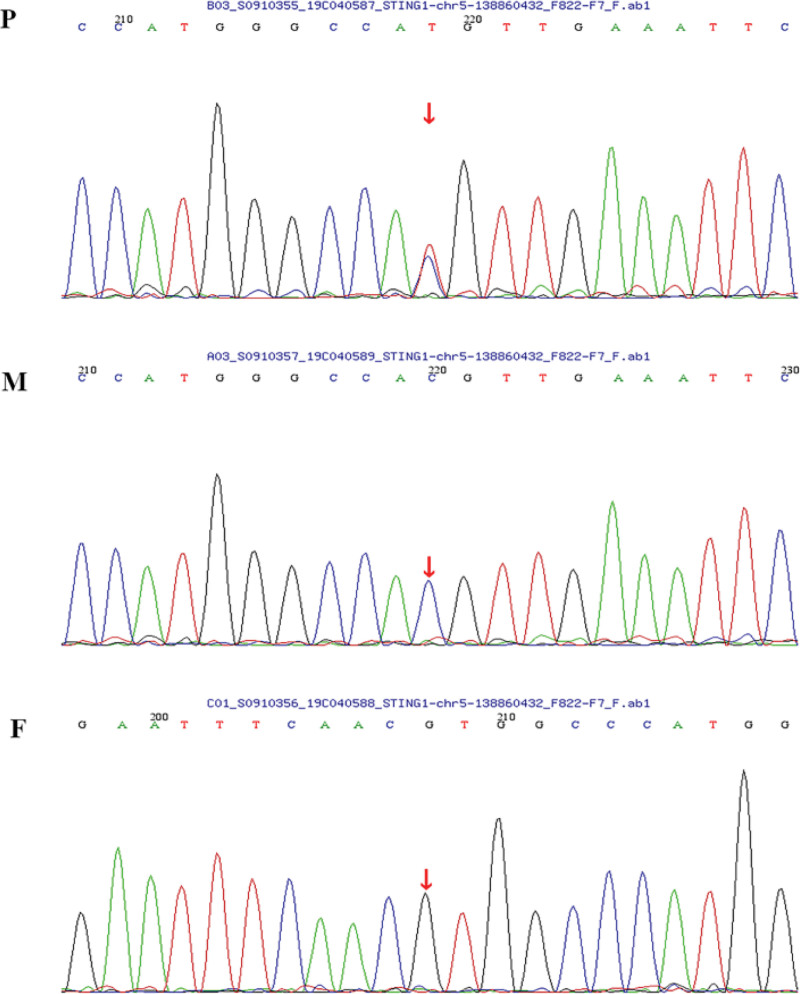
Blood Sanger sequencing of patient. P denotes the case; M demotes the mother of case; F denotes the father of case.

At the time of his admission, the physical examination was normal except for purplish-red non-indurating plaques on the cheeks, nose tip, and auricles (Fig. 1A). Laboratory testing revealed a slightly elevated erythrocyte sedimentation rate (ESR) (27 mm/h) and normal C-reactive protein (CRP) level (1.92 mg/L). Immunological testing revealed elevated IgG (20.1g/L), IgA (1.2 g/L), IgE (>1140 IU/mL), and complement C4 (0.417g/L). The CD8 + T lymphocytes were increased (41.1%) and CD3-CD16 + CD56 + T lymphocytes decreased (4.1%). Antinuclear antibody was 1:100 (reference range, <1:100). In addition, the expression of IFN-regulated genes was higher in the patient’s peripheral blood mononuclear cells (PBMCs) than in healthy controls (Fig. 1D). Renal and hepatic function tests, white blood cell and platelet count, lactate dehydrogenase level were all in normal ranges. Viral and bacterial serology were all normal. The result for bone marrow cytology was negative. Chest HRCT showed ILD characterized by scattered granular and patchy densities with increased shadows (Fig. 1E). Whole-exome sequencing revealed a heterozygous mutation in *TMEM173* (c.463G > A, p.V155M), which was confirmed by Sanger sequencing. The analyses of *TMEM173* of the patient and both parents were given in Figure 2. The patient was ultimately diagnosed with SAVI.

After 8 months of follow-up, the symptom of the boy had no improvement and chest HRCT showed that ILD progressed (Fig. 1F). Then tofacitinib was started at 2.5 mg bid. After 3 months of tofacitinib treatment, the cutaneous manifestations were relieved, and chest HRCT showed improvement of the ILD (Figures 1C and G). However, recurrent oral ulcers and growth retardation persisted. Laboratory testing revealed no significant improvement in the erythrocyte sedimentation rate (ESR), immunoglobulins, or immunophenotype (see Table 1 and Table 2). Antinuclear antibody (ANA) was still positive (1:100). Anti-dsDNA antibody was present (461 IU/mL) and alanine aminotransferase (ALT) was elevated (83U/L).

**Table 1:** Immunoglobulins and complements data of the patient.

	IgG (g/L)	IgA (g/L)	IgM (g/L)	IgE (IU/ml)	C3 (g/L)	C4 (g/L)
1Y	20.1	1.2	0.96	>1140.0	1.16	0.417
19M (pretreatment)	22.9	0.87	0.5	>1200	1.25	0.439
23M (posttreatment)	27.5	1.61	0.9	>1130	1.5	0.475
reference range	3.60–9.20	0.10–0.56	0.38–1.26	0.0–100.0	0.90–1.80	0.10–0.40

**Table 2:** Immunophenotyping of the patient.

	CD19 (%)	CD3 (%)	CD4 (%)	CD8 (%)	CD3 – CD16 + CD56 + (%)
1Y	13.4	80.5	32.9	41.1	4.1
19M (pretreatment)	23.8	64.2	24.8	25.7	5.9
23M (posttreatment)	30.1	65.2	23.2	40.8	3.1
reference range	23–30	56–67	29–40	16–24	8–16

## 3. Discussion

SAVI is a rare auto-inflammatory disease caused by gain-of-function mutations in TMEM173, which encode STING, an endoplasmic reticulum transmembrane protein activated by cyclic guanosine monophosphate-adenosine monophosphate (cGAMP); in turn, this activates IFN regulatory factor 3 (IRF-3), leading to the induction of IFN-β. Mutant STING in SAVI patients leads to increased transcription of the type 1 IFN gene, resulting in JAK activation and phosphorylation of the STAT pathway, which further upregulates the transcription of IFN-responsive genes.^[3]^ Seventeen mutation sites (*N154S, V155M, V147L, V147M, S102P, F209L, F153V, R281Q, R284G, G166E, R284S, G207E, R281W, H72N, G158A, C206Y, F279L*) have been reported in the *STING* gene, with p.V155M being the most prevalent.^[11,12]^

The vasculopathy resulting from vasculitis and endothelial cell death is a hallmark of SAVI, and mostly affects the skin and lungs.^[13]^ Severe vascular-like lesions of the skin, including ulcers and frostbite-like erythema on cold-sensitive areas, are also characteristic.^[14,15]^ Early onset ILD is major concern for patients with SAVI, and ranges from mild ILD without respiratory symptoms to pulmonary fibrosis.^[16,17]^ Most patients also show growth retardation. Arthritis, thyroiditis, retinal vasculopathy, and renal and liver involvement have also been reported in SAVI patients.^[18–23]^

Corticosteroids, disease-modifying antirheumatic drugs, intravenous immunoglobulins, and multiple immunosuppressive therapies are not effective in SAVI patients.^[6]^ Because JAKi inhibits the JAK-STAT pathway and blocks the positive feedback loop of IFN synthesis and release, patients with type I interferonopathies are often treated with a JAKi, including some SAVI patients.^[24–26]^ The first JAKi used in SAVI was ruxolitinib, as reported for 3 children in 2016. Positive effects were noted, including on lung status and cutaneous involvement.^[27]^ Several other reports have described JAKi treatment with ruxolitinib or baricitinib in SAVI; some patients experienced partial symptom relief,^[28–30]^ while others died of acute respiratory failure.^[4,15]^

Tofacitinib was the first JAKi approved for the treatment of autoimmune diseases in humans.^[31]^ It is an effective JAK1 and JAK3 inhibitor that also partially inhibits JAK2 and Tyk2.^[32]^ However, its use for the treatment of SAVI has been limited. In some recent reports, improvements in skin lesions and acral ischemia were noted. Tofacitinib also strongly suppressed IFN in the blood.^[33,34]^ However, 1 study reported that the therapeutic effects of tofacitinib on ILD and rash were limited.^[35]^ Similarly, tofacitinib failed to halt disease progression in a recent study.^[36]^

Our patient was an infant presenting with a facial rash, recurrent oral ulcers, chronic cough, and failure to thrive, who was diagnosed with SAVI at 1 year of age. He was treated with tofacitinib, which improved the cutaneous symptoms and ILD within 3 months. Although tofacitinib exerts its inhibitory effect via the JAK-STAT pathway, we did not find changes in the JAK-STAT pathway in our patient before and after treatment, so cannot clarify the mechanism through which tofacitinib exerts its effects.

SAVI is a difficult-to-treat type I interferonopathy with a high mortality rate due to ulcerating skin lesions and ILD. We hope that JAKi treatment will prove valuable for SAVI patients, but experience with JAKi treatment is limited and further investigation is needed.

## Acknowledgments

This work was supported by grants from the Zhejiang Province Public Welfare Technology Application Research Project (LGF19H100002).

## Author contributions

**Conceptualization**: Xuefeng Xu.

**Data curation**: Xiaorui Fan.

**Supervision**: Qing Zhou.

**Writing—original draft**: Danping Shen.

**Writing—review & editing**: Meiping Lu.
